# Fertility preferences adjusted: reimagining parenthood in response to the uncertainty of infertility

**DOI:** 10.1186/s41118-025-00248-1

**Published:** 2025-05-13

**Authors:** Ester Lazzari

**Affiliations:** 1 Wittgenstein Centre for Demography and Global Human Capital ( IIASA, OeAW, University of Vienna), Dominikanerbastei 16-PSK, 1010 Vienna, Austria

**Keywords:** Infertility, Fertility desires, Fertility expectations, Uncertainty, Australia

## Abstract

Infertility places men and women in a state of considerable uncertainty, characterized by a heightened sense of unpredictability and loss of control. While the experience of such uncertainty might influence individuals’ fertility desires and expectations, so far limited research has explored these relationships. Using longitudinal population-based survey data from Australia, this study examines whether dealing with the uncertainty of infertility prompts men and women to revise their pre-existing fertility preferences. Results indicate that infertility-related uncertainty is a meaningful phenomenon that can illuminate about individuals’ changes in fertility preferences. While fertility expectations are more likely to be adjusted downward in the face of infertility, fertility desires tend to remain mostly unaffected by it in the short-term. The study reflects on the resilience of desires amidst the uncertainty of infertility and considers potential implications for quantitative research on fertility preferences.

## Introduction

The societal trend towards childbearing delay has brought infertility issues at the forefront of factors influencing family formation in contemporary societies. Infertility is medically defined as the failure to conceive or maintain a pregnancy to term after one year or more of regular unprotected sexual intercourse (Zegers-Hochschild et al., 2017). Global lifetime prevalence of infertility is estimated at 17.5% (Cox et al., 2022), indicating that a substantial minority of individuals and couples have been affected by this health issue at some point in their lives. Medical research underscores age as a key factor influencing the biological capacity to reproduce among women and, to a lesser extent, men (ESHRE Capri Workshop Group, 2005; Sartorius & Nieschlag, 2010), while demographic analyses document the increasing trend of couples postponing childbearing to later ages, especially in high-income countries (Sobotka & Beaujouan, 2018). As parenthood continues to be delayed, it is likely that an increasing share of individuals will experience infertility compared to previous generations, increasing the importance of this medical condition in shaping fertility trends (Lazzari et al., 2023a).

Beyond the physical constraint that prevents some from realizing their intended family size (Schmidt et al., 2012), experiencing infertility might also influence people’s fertility desires and expectations by intensifying uncertainty. Scholars have increasingly recognized how uncertainty matters for reproductive decision-making (Kreyenfeld et al., 2012; Trinitapoli & Yeatman, 2018; Vignoli et al., 2020a, 2020b). For instance, demographic research has shown interest in exploring how the impact of various events that increase uncertainty in a person’s life, such as changes in subjective and objective economic situations, relationship status, and age, influence the revision of fertility desires and expectations (Gray et al., 2013; Heiland et al., 2008; Iacovou & Tavares, 2011; Ray et al., 2018; Vignoli et al., 2020b; Wagner et al., 2019). However, how men and women adjust their fertility plans in response to the uncertainty posed by infertility remains poorly understood (Johnson et al., 2018). This gap is surprising given that infertility places individuals in a state of considerable uncertainty, characterized by a heightened sense of unpredictability and loss of control (Greil et al., 2010). In addition, given the considerable and increasing share of couples experiencing infertility at some point in their lives, understanding how fertility plans change in face of this uncertainty is a timely and relevant matter.

Drawing on a longitudinal population-based survey from Australia, this article seeks to contribute to this research area by investigating how men and women revise their fertility desires and expectations in response to infertility. The study uses a self-assessed measure of infertility to address the following research questions: (1) how is the experience of infertility associated with individuals’ fertility desires and expectations? (2) Does infertility lead to a change in their pre-existing fertility desires and expectations? Since previous studies point at gender- and socioeconomic-based differences in how individuals experience and respond to infertility, these outcomes are examined separately for men and women as well as across socioeconomic groups. The analysis is further enriched by examining a subsample of individuals in co-resident relationships (married or cohabiting) because partners’ preferences and characteristics can play an important role in shaping decisions related to childbearing.

## Background

### State of research on the association between self-perceived infertility and fertility preferences

Fertility preferences—a broad term that encompasses a person’s outlook on having children and referring to both concrete intentions or expectations, as well as more general desires and ideals—have been increasingly acknowledged as contextually responsive decisions, with several studies exploring their change in relation to alterations in life-course context. For instance, research has shown that shifts in age, parity, health status, occupational careers, and relationship dynamics—such as entering a new partnership or experiencing a separation—can significantly influence fertility preferences (Gray et al., 2013; Iacovou & Tavares, 2011; Lazzari & Beaujoaun, 2025; Lazzari & Beaujouan, 2025; Liefbroer, 2009; Ray et al., 2018). While no study has specifically examined the evolution of fertility preferences in response to the experience of infertility, some scholars have highlighted the existence of a significant association between infertility and different measures of childbearing preferences. For instance, in a cross-sectional analysis of American women, Shreffler et al. (2016) found that self-identifying as infertile was associated with lower fertility intentions but with higher fertility desires and a greater ideal number of children. Similarly, another cross-sectional study examining the correlates of the intention to have a second child among women attending gynaecology clinics in China found that infertile women expressed a larger ideal family size and placed more importance on childbearing, although they were less confident in achieving their fertility intentions (Lau et al., 2018). These studies suggest a connection between infertility and childbearing preferences. However, the direction of causality remains unclear due to a lack of longitudinal investigations (Johnson et al., 2018).

It is possible that individuals who later experience infertility are initially selected in that they actively sought a pregnancy, resulting in more positive baseline fertility desires compared to those who did not attempt conception. In the face of infertility, these desires might persist due to the strong personal commitment to becoming a parent, further increase as a compensatory response to the challenge of infertility, or decrease if individuals re-evaluate the meaning of childbearing. However, even in scenarios where a decrease occurs, fertility desires might still surpass those of individuals who do not identify as infertile. Regarding fertility expectations, individuals might initially temper their expectations of future childbearing, and these expectations could remain relatively low after their failure to conceive. It is also possible that those initially holding relatively high fertility expectations revise them downwards following challenges to conceive. All these scenarios are consistent with the finding that women with infertility exhibit higher fertility ideals and lower fertility expectations compared to fertile women (Lou et al., 2018; Shreffler et al., 2016). An examination of individuals’ fertility preferences before and after the experience with infertility is essential to shed light on these mechanisms.

Another important research gap concerns gender differences in the context of infertility (Almeling, 2015; Johnson et al., 2018). While our understanding is limited regarding the link between fertility impairments and childbearing preferences, there is even less knowledge about how this association varies between genders as no study so far has examined how fertility preferences change among men who have experienced infertility. Existing literature indicates that individuals differ in how they experience and respond to infertility based on gender (Greil et al., 2010; Lazzari et al., 2022b; Passet-Wittig et al., 2020; Ying et al., 2015). This discrepancy might be attributed to differences in normative expectations, biological processes, and sensitivity to pregnancy-related experiences. Given that perceptions and consequences of infertility can differ significantly between men and women, it is relevant to explore gender-specific associations in the relationship between infertility and fertility preferences.

An individual’s socioeconomic status is also likely to influence the degree to which they believe they can mitigate the consequences of infertility. For example, those with greater financial resources might feel more confident that they can access assisted reproductive technology (ART) treatments to address their medical condition, which could in turn shape their response to infertility (Greil et al., 2010). This aligns with research showing that the use of infertility services varies due to systemic barriers (Inhorn, 2020). Even in countries with supportive public funding for ART, such as Australia, significant disparities in access to treatment persist (Lazzari et al., 2022a). As a result, the level of uncertainty brought about by infertility might vary depending on a person’s economic circumstances, which might mediate the association between infertility and fertility preferences.

As a condition that affects both partners in a couple, responses to infertility are likely influenced not only by individual factors, but also by the couple-level context and the characteristics of one’s partner (McQuillan et al., 2021; Lazzari et al., 2022b). For example, responses to infertility might be mediated by relationship satisfaction, which has been found to have a protective effect against various types of emotionally stressful events (Røsand et al., 2012), and might also vary depending on the extent to which childbearing is important to the partner (Duvander et al., 2020; Testa & Bolano, 2021).

### The uncertainty of infertility

Acknowledging the role of uncertainty is crucial for understanding the evolution of fertility preferences (Bachrach & Morgan, 2013; Gray et al., 2013; Heiland et al., 2008; Ní Bhrolcháin & Beaujouan, 2015; Vignoli et al., 2020a). While the influence of economic uncertainty on fertility choices has received considerable attention (Kreyenfeld et al., 2012; Vignoli et al., 2020b), other sources of uncertainty—such as those stemming from negative reproductive experiences like pregnancy loss or fertility impairments—remain relatively unexplored (Ní Bhrolcháin & Beaujouan, 2015). The lack of research on these topics highlights the need to bridge the often-separated biological and social dimensions of fertility decision-making (Almeling, 2015; Johnson et al., 2018).

Research indicates that feelings of uncertainty and loss of control often accompany infertility (Benyamini et al., 2008; Clarke et al., 2006; Greil et al., 2010). While reproduction inherently involves uncertainty, infertility further amplifies this unpredictability, making it more challenging for individuals to foresee their journey into parenthood and formulate strategies. As noted by Zucker (1999), this heightened uncertainty stems from infertility’s dual nature: is both chronic, spanning months or even years, and unplanned, as it arises unexpectedly.

Uncertainty can be experienced on multiple fronts. One major source relates to the possibility and timing of achieving parenthood. The medical definition of infertility—the failure to conceive after a medically determined period—implies a reduced likelihood of conception rather than an absolute inability to have children. Natural conceptions, for instance, are not uncommon among women who previously sought ART treatment (Thwaites et al., 2023). In line with this medical understanding, most infertile couples do not view parenthood as completely out of reach (Greil, 1991). The potentially reversible nature of infertility is further emphasized by studies indicating that individuals do not consistently identify as infertile (Passet-Wittig et al., 2020), and that reporting infertility does not necessarily correspond with having fewer children (Greil et al., 2024). Taken together, these elements highlight infertility as a condition whose resolution remains fundamentally uncertain.

The path toward parenthood introduces another layer of unpredictability. Alternative routes such as ART or adoption offer possibilities, but they also present ambiguous outcomes. For example, for some the use of ART may increase a sense of control over infertility, while for others it may simply perpetuate their state of uncertainty (Letherby, 2002; Miller, 2004; Yu et al., 2021).

Uncertainty extends beyond reproductive outcomes to issues of identity, as infertile individuals may struggle with a discrepancy between their desired social role of being a biological parent and their current status as infertile. While they cannot confidently assume they will become parents, they neither fully embrace childlessness, a state that Greil (1991) describes as “spoiled identity”. As a result, they may feel uncertain about how to behave and what to hope for. Infertility may also indirectly increase uncertainty regarding future reproduction by destabilizing key relationships tied to one’s identity as a biological parent (Loftus & Namaste, 2011). Research suggests that the quality and stability of romantic partnerships might depend on the ability to fulfil the parent role in the future (Luk & Loke, 2015; Pelikh et al., 2024).

### The instability of fertility preferences

The formation of fertility intentions is often conceptualized as a dynamic process that involves the integration of desires with an assessment of reality and perceived situational constraints. Miller and Pasta’s traits–desires–intentions (TDI) model (1993, 1995) proposes that fertility desires are relatively stable and represent a person’s ideal vision regarding childbearing in the absence of situational constraints. In contrast, fertility intentions may vary in response to current circumstances and are rooted in a commitment to act. Another popular framework for conceptualizing fertility intentions, the Theory of Planned Behaviour (TPB) (Ajzen & Klobas, 2013; Fishbein & Ajzen, 2010), also underscores the influence of individual situations and perceived behavioural control in the formation of fertility plans, which are seen as the result of rational choice.

An alternative perspective to rational choice theory is offered by the Theory of Conjunctural Action (TCA) (Johnson-Hanks et al., 2005) and the Cognitive Social Model (CSM), which draw on insights from cognitive science. These models caution against overemphasizing the role of explicit intentions in shaping behaviour recognizing that childbearing decisions are also influenced by emotional associations and mental models or schemas (Bachrach & Morgan, 2013; Johnson-Hanks et al., 2011). Consistent with this framework, many empirical analyses have shown that childbearing ideals are not static across the life course (Heiland et al., 2008; Müller et al., 2022; Ray et al., 2018) and can change in response to uncertain life circumstances (Gray et al., 2013; Lazzari et al., 2023b; Trinitapoli & Yaetman, 2018) raising questions about their fixed nature.

In the Narrative Framework uncertainty assumes a central role (Vignoli et al., 2020a, 2020b). While individuals are influenced by structural and contingent constraints in the formulation of their expectations, they also have the capacity to envision alternative scenarios that are not merely the result of current and past conditions (Vignoli et al., 2020b). An increase in uncertainty may have a negative influence on fertility plans, however people may still maintain positive imaginaries related to family and the emotional meaning of parenthood may remain unaltered by the uncertainty they experience. As a result, their fertility strategies are not only determined by current and past experiences, but also shaped by imagined futures (Vignoli et al., 2020a). This is a key aspect of the Narrative Framework that allows to reconcile differences between expectations, which are based on current constraints, and individuals’ aspirations of future selves, which are influenced by their normative value orientations (Vignoli et al., 2020a).

### Aims and hypotheses

This study has two primary aims. First, it seeks to investigate the cross-sectional association between infertility and fertility desires and expectations. Drawing on findings from previous research focusing on women (Lau et al., 2018; Shreffler et al., 2016), it is anticipated that there will be a negative association between infertility and fertility expectations (Hypothesis 1a), as well as a positive association between infertility and fertility desires (Hypothesis 1b) at each examined time point. The second aim of this study is to explore whether the experience of infertility leads to alteration in individuals’ fertility desires and expectations from a longitudinal perspective. Considering infertility as a situational constraint, it is hypothesized that expectations will be revised downward in response to infertility (Hypothesis 2a). While existing evidence shows that desires can adapt to life circumstances and may change in response to uncertainty, the extent to which they remain flexible in the face of infertility remains unclear. Previous research suggests that various conditions may have different impacts on individuals’ fertility desires (Grey et al., 2013; Trinitapoli & Yeatman, 2018). The likelihood of these impacts may depend on the strength of individuals’ initial desires before experiencing the condition and the extent to which the condition itself can reshape their representations of future selves (Vignoli et al., 2020a). Thus, no specific Hypothesis 2b is formulated regarding the association between changes in fertility status and changes in desires.

Social and economic conditions may influence these responses. Individuals with higher socioeconomic status may experience lower levels of uncertainty due to better access to ART. In contrast, those with lower socioeconomic status may face greater uncertainty regarding the implications of infertility. Based on Hypotheses 1a and 1b, this uncertainty is expected to translate into stronger negative adjustments in their fertility expectations (Hypothesis 3a). No specific hypothesis is proposed regarding changes in desires (Hypothesis 3b).

Finally, existing literature suggests that the impact of infertility is often more emotionally stressful for women than for men (Greil et al., 2010; Ying et al., 2015), with women having a higher awareness of fertility issues within the couple (Lazzari et al., 2022b), and infertility being a more salient factor for women’s identity than for men’s (Ying et al., 2015). Consequently, it is hypothesized that the associations found between infertility and desires (or expectations) in both cross-sectional and longitudinal analyses will be in a similar direction but more pronounced for women than for men (Hypothesis 4).

### Study context

Parenthood remains a central life goal for most Australian men and women (Holden et al., 2005; Holton et al., 2011; Qu, 2020). Among those wishing to have a child, survey data indicate that the social norm of having two children has remained largely unchanged over the past two decades (author’s calculations based on the Household Income and Labour Dynamics in Australia (HILDA) Survey). However, completed family size has consistently declined (Gray & Lazzari, 2023), with recent trends in period fertility rates suggesting that current generations in their childbearing years will eventually have fewer children than previous cohorts (Qu & Baxter, 2023). The increasing mean age at birth likely contributes to the observed fertility gap between intended and achieved fertility. Statistics on the age of first-time mothers reveal that, in 1991, approximately a quarter of women had their first child at age 30 or older, while by 2020, this figure had risen to over half (Qu & Baxter, 2023). The trend towards fertility postponement is also evident in survey data, which show a rising proportion of women expressing a desire to have a child past the age of 35 (Lazzari et al., 2023a).

Since the biological capacity to conceive declines with age, infertility prevalence has likely increased. While the association between age and infertility is more relevant for women, reproductive capacity also diminishes with age in men (ESHRE, 2005; Sartorius & Nieschlag, 2010). Moreover, since infertility affects couples, men are subject to their partner’s stricter age deadline. Recent estimates suggest that 16% of heterosexual couples in Australia experience difficulties conceiving (Lazzari et al., 2022b). The importance of infertility issues in the country is further underscored by the rapid rise in the utilization of ART treatments, which have become increasingly important in supporting the family-building plans of couples with infertility (Lazzari et al., 2023a). Australia’s universal healthcare system, Medicare, provides supportive funding for ART without imposing restrictions on either male or female age, parity, and the number of treatments previously subsidized. Typically, general practitioners serve as the first point of contact for individuals with infertility concerns, as patients cannot self-refer to fertility clinics. Women are about three times more likely than men to consult medical providers for infertility concerns (Chambers et al., 2019). Additionally, women from advantaged socioeconomic backgrounds are more likely to seek medical consultations for infertility than women from less advantaged backgrounds, although the same trend has not been observed among men (Chambers et al., 2019).

### Data and methods

#### Sample

The data for this study are sourced from two waves of the Household Income and Labour Dynamics in Australia (HILDA) survey. HILDA is a nationally representative panel study of Australian households, collecting annual data on a broad range of demographic, health, and socioeconomic indicators. The first wave of the HILDA survey was collected in 2001 and included more than 13,000 participants (Summerfield et al., 2022). The household response rate in the first survey was 66%, comparing favourably with other household-level surveys (Wooden et al., 2002). Response rates for subsequent waves remained above 90% (Summerfield et al., 2022).

The choice of using the HILDA survey data stems from its unique ability to provide longitudinal information about the self-assessed infertility status of men and women. Another strength of this survey is the inclusive sampling approach, which involves asking questions about fertility preferences to all reproductive age individuals, irrespective of whether they have experienced difficulties conceiving. This inclusivity is remarkable as infertile respondents are often wrongly excluded from answering fertility-related questions, under the incorrect assumption that those experiencing infertility lack fertility preferences (Passet-Wittig et al., 2020).

Questions related to infertility and fertility preferences (desires and expectations), the primary variables of this study, were administered through face-to-face interviews with all adult household members who were of reproductive age. Due to data availability, the analysis focuses on expectations rather than intentions. While intentions and expectations may have conceptual differences (Rackin and Bachrach 2016), empirical studies suggest that they function similarly (Gemmill, 2019; Ní Bhrolcháin & Beaujouan, 2015)^1^. Information regarding the infertility status of respondents was first collected in 2005 as part of a rotating fertility module (administered every 3 or 4 years from Wave 5 onwards). For the purpose of this analysis, the two most recent waves of data were used, collected in 2015 (referred to as Time 1 or T1) and 2019 (referred to as Time 2 or T2).

The analytical sample of this study consists of individuals representing the reproductive age population—specifically, women aged between 18 and 45 and men aged between 18 and 50 in T1. These age categories were determined by the fact that questions about infertility, the main explanatory variable, were collected for women aged below 50 and men aged below 55. Respondents are included in the analysis regardless of their relationship status. While it may be more difficult for individuals in non-coresidential unions to meet the medical criteria for infertility (one year of regular unprotected intercourse), they may still identify as infertile based on their reproductive experiences, attempts to conceive, and perceived difficulties in achieving pregnancy. After removing 244 respondents (3.6%) due to missing values in one or more explanatory variables, the final analytical sample for the cross-sectional analysis totalled 6520 respondents. For the longitudinal analysis, the sample was further restricted to only include respondents who were present at follow-up 4 years later in T2 (73.4%)^2^. In addition, 415 respondents who were infertile at T1 were excluded, along with 241 respondents (6.1%) who had missing values in either of the two waves. This resulted in a final analytical sample of 3,702 individuals for the longitudinal analysis. Additional analyses examined 777 heterosexual couples where the woman was aged 18 to 45 and the man was aged 18 to 50 in the first wave. To be eligible for inclusion in the couple sample, respondents needed to be either married or in a cohabiting relationship with a partner of the opposite sex at T1 and had to remain in that relationship at follow-up.

#### Concepts and measures

This study investigates how the experience of infertility influences women’s fertility preferences using two indicators: fertility desires and fertility expectations, capturing ideal and anticipated life-course patterns, respectively. Individual desires were assessed through the question: ‘*How do you feel about having a child (more children) in the future?*’. Answers were measured on an 11-point Likert scale, where 0 indicated ‘*definitely would not like to have children*’ and 10 indicated ‘*definitely would like to have children*.’ Individual expectations were assessed through the question: ‘*How likely are you to have a child (more children) in the future?*’ and measured on an 11-point Likert scale, where 0 indicated ‘*very likely to have children*’ and 10 indicated ‘*very unlikely to have children*.’ Questions on fertility preferences were not asked to sterile respondents; therefore, analysing fertility preferences within this group was not possible.

The main explanatory variable is a binary measure indicating whether respondents experienced infertility between T1 and T2. This variable is derived from the question: ‘*Based on medical advice, do you know of any physical or health reason that would make it difficult for you (and/or your partner) to have [children / more children]?*’. Answering options were ‘*yes*’, ‘no’, and ‘*don’t know*’. As very few respondents gave ‘*don’t know*’ as an answer, these cases were excluded from the analysis. This question was not asked to respondents who were sterile (they had a permanent inability to conceive) and those who were either pregnant or had pregnant partners.

The recognition of infertility may vary, with some women identifying as infertile despite not meeting the medical criteria and others not self-identifying as having a fertility problem even if they qualify as medically infertile (Greil et al., 2014; White et al., 2006). Given that this study seeks to understand how the lived experience of infertility shapes fertility preferences, it is reasonable to focus on questions capturing perceptions rather than objective medical conditions. However, it is important to consider that, since the definition of infertility in HILDA is conditional on having received medical advice, it may fail to capture individuals who perceive themselves as infertile but report otherwise due to a lack of consultation with a medical professional. This underestimation is likely more pronounced among individuals from less advantaged socioeconomic backgrounds or those living in remote areas, as they are less likely to seek medical advice for infertility (Chambers et al., 2019). Nonetheless, people are generally more inclined to consult a professional when they desire parenthood and already identify as infertile (Greil et al., 2010), suggesting that infertility as measured in this context remains shaped by social and behavioural processes.

Several demographic factors and individual characteristics are considered as control variables. Demographic factors include the respondent’s age group, parity (categorized as 0, 1, 2, or 3 or more children), and relationship status (single, cohabiting, and married). For longitudinal analyses, two categories of change in relationship status were created: (1) transition to singlehood (from living with a partner); and (2) transition to living with a partner (from being single). Longitudinal models also account for whether respondents gave birth between waves, which may alter their preferences for future children.

Individual characteristics include measures of socioeconomic status and background variables. Measures of socioeconomic status comprise educational attainment (classified as lower secondary or less, upper secondary, and higher education) and economic situations, assessed using an item that asked about respondents’ satisfaction with finances in relation to their current needs and financial responsibilities. The economic situation variable distinguishes between respondents who were: very satisfied, reasonably satisfied, and dissatisfied with finances. In the longitudinal analysis, the variable for change in economic situation is coded as ‘more satisfied with finances’ for respondents who report an increase in financial satisfaction between waves, and ‘less satisfied with finances’ for those whose satisfaction decreases. The decision to use a subjective measure of financial wealth stems from the understanding that subjective indicators of one’s economic circumstances may be better proxies for the level of economic uncertainty experienced by the individual than objective financial situations (Vignoli et al., 2020b).

Turning to the background characteristics, the migration and indigenous status variable groups respondents into three categories: ‘non-migrant’, referring to individuals born in Australia to both Australian parents; ‘migrant’, encompassing those born abroad or born in Australia to at least one non-Australian parent; and ‘Indigenous Australian’, including respondents who reported to be of Aboriginal or Torres Strait Islander origin. Area of residence is classified into three categories, reflecting whether individuals dwelled in major cities, inner regional areas, or outer regional, remote, and very remote areas.

Given the infrequent occurrence of changes in educational attainment and area of residence during the two analysed time periods, these variables are treated as fixed. To assess the robustness of the results, supplementary analyses were performed by treating these variables as time-varying (not shown but available upon request). The outcomes reveal no substantial differences from the results presented in this paper.

#### Analytic strategy

The sample strategy created a group of people who remained fertile throughout the entire period (the ‘control’ group) and a group of individuals who reported to be infertile only at T2, and hence transitioned into the infertility status (the ‘treatment’ group). Since the event of experiencing infertility is not randomly assigned, this cannot be considered as a true experimental design. To adjust for potential initial differences among individuals that did and did not experience infertility between waves, ordinary least square regressions were estimated using the regressor variable method (Allison, 1994). With this approach, the effect of infertility on fertility goals at T2 is estimated as a function of fertility goals at T1, time-invariant characteristics measured at T1, and between-wave changes in life course variables. The regressor variable method was preferred over the change score method, as there may be a positive association between fertility desires at T1 and identifying as infertile at T2. Research suggests that individuals with stronger fertility desires are more likely to forgo contraception or actively attempt to conceive, which could increase the likelihood of experiencing infertility (Gemmill et al., 2021; Passet-Wittig et al., 2020; Polis & Zabin, 2012).

In its basic form, the model is presented in Eq. 1, where *Y*_*it*_ is the fertility preference of respondent *i* at follow-up and *Y*_*it*−1_ is the fertility preference of respondent *i* measured in the previous wave. Between the two time points, the respondent may (*X*=1) or may not (*X*=0) experience infertility. The occurrence of infertility is identified using data collected at follow-up in T2. The aim of this model is to assess whether the occurrence of infertility (*X*_*it*_) affects fertility preferences at T2 (*Y*_*it*_), holding constant the possibility that respondents experiencing the event had a different mean on *Y*_*it*−1_ than those who did not experience the event:

(1) $$Y_{it}=b_{0}+b_{1}X_{it}+b_{2}Y_{it-1}+e_{i}.$$

Equation 1 can be expanded to include time-varying independent variables to provide an estimate of the effect of a change in other life events that can also influence fertility preferences, and time-invariant variables measured at T1 that do not change over time. Time-varying variables used in the models include parity, relationship status, and satisfaction with finances. Time-invariant variables include age, education, migration and indigenous status, and area of residence.

I begin by presenting cross-sectional analyses of the correlates of fertility desires and expectations using data collected at T1. I first provide an overview of the sample and present descriptive statistics of all study variables by infertility status. Next, I conduct ordinary least square regression (OLS) analyses to investigate the predictors of fertility desires and expectations. These analyses are replicated using data collected at T2. Results from these additional analyses (Tables S1, S2, and S3 in the Online Appendix) did not reveal substantial differences from the findings presented in this paper. Then, for the longitudinal analysis, I first present descriptive evidence of changes in fertility desires and expectations between T1 and T2 for those who never experienced infertility and those who transitioned from being fertile in T1 to experiencing infertility in T2. Finally, I investigate the extent to which fertility desires and expectations change in response to transitioning to experiencing infertility, while controlling for other relevant changes in life-course context using the regressor variable method.

### Analyses of individuals in a co-resident relationship

Research highlights how men and women are influenced by their partner’s characteristics when revising their childbearing plans (Berrington, 2004; Testa & Bolano, 2021). Therefore, part of the analysis considers the impact of partners’ and relationships’ characteristics on changes in fertility preferences among a subset of individuals in heterosexual co-resident unions (married or cohabiting). A new and more detailed model is estimated that specifically considers the role played by the partners’ initial fertility desires (or expectations), education, and age difference with the respondent. Relationship-specific variables, such as the type of union and relationship satisfaction, are also considered, as these may be important mediating factors influencing the relationship between infertility and fertility preferences. These analyses test the same hypotheses as the main analysis—the extent to which fertility desires and expectations vary in response to infertility.

## Results

### Association between fertility preferences and infertility at a single time point

**Fig. 1:**
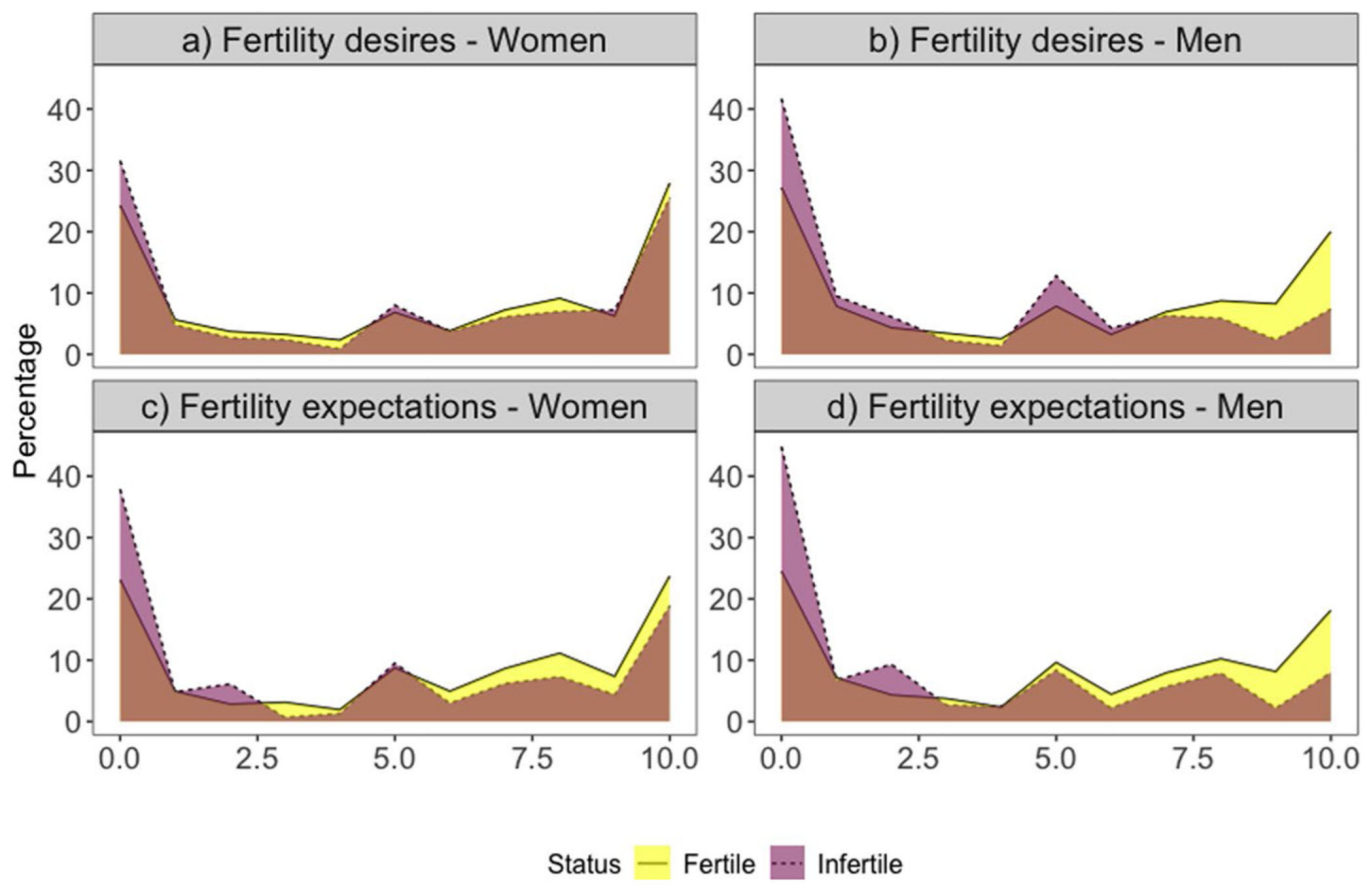
Percentage distribution of fertility desires and expectations in T1 by self-reported infertility status. *N* = 3329 women and 3191 men interviewed in wave 15 with non-missing data on dependent and independent variables. Values are weighted. The corresponding numerical values can be found in the online Appendix (Table S4). Source: HILDA survey, Wave 15, release 21

Figure 1 illustrates the distribution of fertility desires and expectations by self-reported infertility status using data from T1 (means and exact values of the distributions are reported in Table S4 in the Online Appendix). Responses to questions about fertility desires and expectations tend to cluster at the two extremes of the distribution. Infertile men are more likely to express lower desires and expectations compared to their fertile counterparts. For instance, 42% of infertile men have a fertility desire score of zero, and 45% have a fertility expectation score of zero, compared to 27% and 25% of fertile men, respectively. Among women, differences in fertility desires are less pronounced, though fertile women appear slightly less likely to desire a child, which is in contrast with previous studies (Shreffler et al., 2016). However, infertile women are clearly more likely to report lower expectations—just under 40% of infertile women express the lowest expectation to have a child compared to only 23% of fertile women. Data from T2 show a similar pattern, as detailed in the Online Appendix (Table S1).

Table 1 provides a comparison of descriptive statistics for all study variables by infertility status. The samples of fertile and infertile respondents differ mostly in their age distribution, with infertile men and women more likely to be older. Infertile respondents are also more likely to have only one child but are less likely to be childless, which may reflect a stronger commitment to becoming parents among those who self-identify as infertile (Shreffler et al., 2016). Another difference of the infertile sample compared to the fertile sample is the higher probability of being in a relationship and it is especially evident when looking at the male sample, with only 12.3% of men who self-reported to be infertile being single compared to 43.6% in the fertile group. This pattern suggests that men might be less aware of fertility issues unless they experience them in a couple-level context. It could also reflect the way in which infertility is measured in HILDA, where the question refers to the fertility of both partners, potentially leading partnered individuals to report infertility more frequently. Fertile women are more likely to be highly educated, while the opposite trend is observed among men. Finally, financial dissatisfaction appears more common among infertile respondents of both sexes. Similar trends are observed in the T2 data (Table S2 in the Online Appendix).

To assess the association between infertility status and fertility desires and expectations, net of demographic, socioeconomic, and background characteristics, results from linear regression models are presented in Table 2. I present results from T1, but patterns from T2 are substantially similar (Table S3 in the Online Appendix). Distributions of the independent variables in Table 2 are provided in Table 1. In the unadjusted Model 1, there is no significant relationship between fertility desires and self-reported infertility status among women. However, once respondents’ characteristics are accounted for in Model 2, there is a positive association between infertility and fertility desires (this association is not statistically significant for the T2 sample, although it remains in the expected direction). This result is in line with previous studies and consistent with Hypothesis 1b. Among men, the association between infertility and fertility desires is not statistically significant in Model 2. Moving to expectations, the unadjusted Model 1 reveals that men and women who self-identify as infertile are significantly more likely to have low fertility expectations compared to those who do not. After controlling for respondents’ characteristics in Model 2, the negative association between infertility and fertility expectations becomes less pronounced, but it remains statistically significant. This suggests that this negative association is not simply driven by differences in groups characteristics, such as age and parity. This result is also consistent with previous literature and in line with Hypothesis 1a. Two additional models were fitted to examine the potential impact of gender on fertility desires and expectations (not shown). In contrast to Hypothesis 4, results from these models indicate the absence of gender-specific effects.

### Change in fertility preferences in response to infertility

**Fig. 2:**
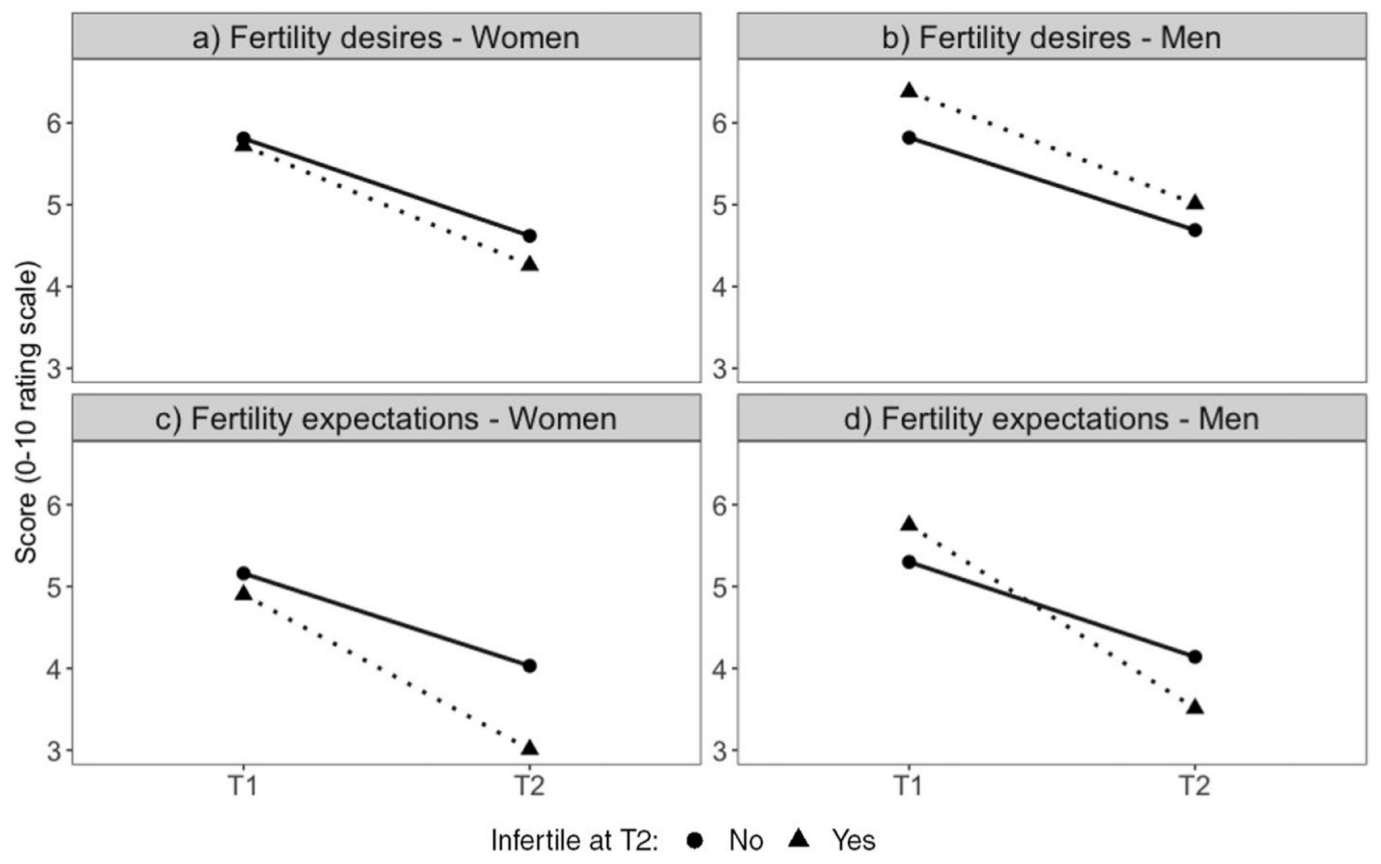
Mean change in fertility desires and expectations between T1 and T2 for respondents who were fertile in T1 by their infertility status in T2. Note: *N* = 1857 women and 1845 men interviewed in wave 15 and 19 with non-missing data on dependent and independent variables. The corresponding numerical values can be found in the online Appendix (Table S5). Source: HILDA survey, Wave 15 and 19, release 21

In the longitudinal analysis, I examine changes in fertility desires and expectations between T1 and T2 (3 years apart) among individuals who initially identified as fertile in T1. During this interval, 11.3% of women and 5.5% of men transitioned into the category of infertile, whereas 88.7% and 94.5% continued to identify as fertile at T2 (Table 3). Figure 2 reports the mean fertility desires and expectations at both T1 and T2 for the two groups: individuals who consistently identified as fertile in T1 and T2 and those who transitioned to self-identify as infertile by T2. The reported values represent the mean scores for the variables of fertility desires and expectations, according to respondents’ T2 infertility status.

In general, Fig. 2 indicates that, on average, women who did not experience infertility between the two waves reported similar fertility desires and expectations at T1 to those who later transitioned to self-identifying as infertile. By contrast, men who later identified as infertile expressed higher fertility desires and expectations at T1 than those who remained fertile, on average. However, these differences do not appear to be statistically significant, as determined by two-sample t-tests (Table S5). In other words, there were no meaningful disparities in the desires and expectations of the two groups prior to the identification of infertility. By T2, men and women in both groups adjusted their fertility desires and expectations downward. This adjustment was relatively more pronounced in relation to expectations rather than desires and among individuals who experienced infertility (Fig. 2). Results from the two-sample t-tests indicate that, for women with infertility, the downward revision of fertility expectations was significantly larger than that of their counterparts who did not experience infertility during the 3-year period (Table S5).

To address potential bias due to time-varying factors, Table 4 presents multivariate models accounting for events that may have intervened in the lives of respondents between waves of data collection and influence their fertility preferences. These events include whether respondents had a child between waves as well as alterations in relationship status and changes in financial satisfaction. Additionally, the models in Table 3 incorporate a control for the respondent’s fertility preference a T1. The inclusion of this variable is crucial as it adjusts for potential variations in initial fertility desires or expectations among the group of respondents who experienced infertility and those who did not. Descriptive statistics of the independent variables in these models are presented in Table 3.

Table 4 shows that, after adjusting for all control variables and between-wave changes in Model 2, the experience of infertility is associated with a downward revision of expectations among both men and women (Hypothesis 2a), although it is not linked with a significant change in fertility desires (Hypothesis 2b). Clearly, having a child has a strong influence on the revision of fertility preferences, as well as a change in relationship status, with those forming new partnerships being significantly more likely to desire and expect to have a child compared to those who did not change their relationship status. However, net of these events, transitioning from the fertile to the infertile status also brings relevant implications for fertility expectations. On the other hand, the insignificance of a change in infertility status for desires points at the stability of ideals compared to expectations in face of infertility.

Supplementary models using interaction analyses between infertility status and each one of the other independent variables were also performed. Results from these analyses did not reveal any significant pattern. In particular, the idea that lower socioeconomic status, as captured by education and satisfaction with finances, would lead to a stronger revision of fertility expectations (Hypothesis 3a) was not supported and no evidence was found for an effect of socioeconomic status on the revision of fertility desires (Hypothesis 3b). Additional models with an interaction term between gender and infertility status indicated that gender-specific effects were also not statistically significant (Hypothesis 4).

### Additional analysis

To provide a more comprehensive understanding of the role of infertility in shaping fertility preferences, the longitudinal analysis was repeated among a subsample of individuals in co-resident relationships (Table 5). This analysis allows for the inclusion of the partner’s fertility preferences, a key variable associated with an individual’s desire and expectation to have a child. Excluding those without a co-resident partner strongly reduced the sample size. However, coefficients did not substantially change. By and large, the findings from these analyses confirm what was found in the main sample : men and women with an experience of infertility are more likely to revise their fertility expectations downwards, while their fertility desires do not significantly change in response to infertility. Indeed, after considering additional situational factors relevant to partnered individuals, such as the partner’s fertility preferences in T1, the experience of infertility remains a relevant factor shaping couple’s expectation to have a child, but not their desire.

### Study limitations

There are some limitations to this study. First, infertility is not a stable trait (Passet-Wittig et al., 2020), and it is possible that respondents’ assessment of their infertility status changed multiple times between T1 and T2. It is also possible that individuals who self-identified as fertile in T1 had experienced infertility before, and so their fertility expectations depend on the accumulation of their infertility experiences over time. Measuring self-assessed infertility status only at two points in time may not adequately capture these changes. Second, the interpretation of the results is complicated by the ambiguity of whether respondents interpreted the question on infertility as referring to themselves as individuals or as members of a particular couple. Depending on how the question was intended, it may influence the association with fertility preferences. Third, some respondents who have experienced difficulty conceiving may have undergone infertility treatment, which may have influenced the observed association between infertility and fertility preferences. For example, the lack of clear-cut results regarding the mediating effect of socioeconomic factors could be attributed to the fact that respondents with greater financial resources were more likely to have pursued infertility treatments compared to those with fewer resources (Harris et al. 2016; Lazzari et al., 2022a). Consequently, their perceptions regarding the ease or difficulty of overcoming infertility may differ from those who did not seek treatment. Particularly for individuals who have undergone unsuccessful treatment, their expectations regarding future childbearing may be more pessimistic, despite having greater financial means to pursue parenthood goals. Unfortunately, the HILDA survey does not provide information regarding whether respondents have undergone ART treatment, and, thus, this mechanism cannot be disentangled in the present study. Future research should attempt to incorporate this aspect when examining the association between infertility and the change in fertility preferences.

## Discussion and conclusion

Due to delayed childbearing, the proportion of individuals and couples experiencing difficulty conceiving has increased in recent decades, potentially impacting their reproductive plans and strategies. However, despite the increasing prevalence of infertility, little is known about its influence on people’s fertility desires and expectations. This study contributed to address this research gap by examining how the experience of infertility is associated with men’s and women’s fertility preferences at a specific point in time, as well as weather the encounter with infertility prompts a revision in individuals’ pre-existing fertility preferences over a 3-year period.

Using a population-based survey from Australia, the cross-sectional investigation revealed that, in line with Hypothesis 1a, infertility was associated with lower fertility expectations among both men and women. Partial support for Hypothesis 1b was found, as fertility desires were higher among infertile women, although no significant association was observed among men. These findings corroborate previous cross-sectional studies demonstrating similar associations among women (Lou et al., 2018; Shreffler et al., 2016). On the other hand, the lack of a significant association among men may indicate gender differences in coping mechanisms and emotional responses to infertility. The longitudinal analyses provided robust evidence for Hypothesis 2a, indicating a significant downward revision in fertility expectations following the experience with infertility. These results held when the analyses were restricted to individuals in coresidential relationships controlling for key variables influencing one’s fertility preferences, such as the partner’s wish for a child. In contrast, infertility did not appear to affect desires (Hypothesis 2b). Contrary to Hypothesis 3, which posits that the influence of infertility on fertility preferences would vary depending on a person’s socioeconomic status—seen as a lens through which the infertile condition may be perceived as more or less manageable—the results indicate that socioeconomic factors do not significantly moderate this relationship. In interpreting these findings, it is important to consider the supportive policy environment for ART in Australia. In countries with lower accessibility to ART services, infertility may impose stronger constraints on the reproductive prospects of couples with lower socioeconomic status (Inhorn, 2020). An alternative explanation for the lack of differences is that individuals may be at different stages of the help-seeking process, with those with greater means more likely to have used ART and to be better informed about their chances of overcoming infertility. This, in turn, may influence their expectations about the likelihood of having a child relative to those with fewer resources.

Interestingly, despite literature suggesting that infertility may induce greater distress in women compared to men (Greil et al., 2010; Ying et al., 2015), Hypothesis 4, positing a more pronounced effect of infertility on women’s fertility preferences than men’s, was not supported in any of the analyses. This unexpected result may reflect shifting gender norms around parenthood or a growing emotional investment in fertility among men, particularly those in committed relationships. It is also possible that men who are aware of their infertility may already represent a more selective, engaged group, reducing observable gender differences.

Results from the longitudinal analyses align with theoretical perspectives suggesting that certainty is a prerequisite for the formulation of an intention (Ajzen & Klobas, 2013) and that fertility expectations are influenced by current constraints (Miller & Pasta, 1993, 1995; Vignoli et al., 2020a). By contrast, the uncertainty introduced by infertility does not substantially alter fertility desires—at least in the short-term. Although desires are increasingly conceptualized as dynamic and influenced by uncertainty (Gray et al., 2013; Trinitapoli & Yeatman, 2018), this study shows that they tend to remain stable in the face of a biological constraint such as infertility. This suggests that, in the short-term, the emotional meaning of parenthood and individuals’ imagined reproductive future (Vignoli et al., 2020a) might not change following an episode of infertility. For infertile men and women, fertility desires may reflect a deeply ingrained psychological commitment to parenthood that persists even when the ability to conceive becomes uncertain.

These findings hold implications for scholars interested in the study of fertility preferences, suggesting that individuals experiencing difficulty conceiving approach fertility-related questions differently from those who do not. While their fertility desires may change in a similar fashion over a short period of time, their fertility expectations decline more. Their state of uncertainty regarding the possibility of having a child may prevent them from reporting positive fertility expectations, despite actively trying for a child and maintaining a strong desire for parenthood. This underscores the importance of including information about respondents’ infertility status in quantitative research on fertility preferences to account for the instability in reproductive expectations over time and differences between desires and expectations. Otherwise, these variations may be misinterpreted as irrational or indicative of unstable reproductive plans.

The present investigation also sheds light on fertility decision-making under conditions of uncertainty. The findings support the idea that a current uncertain situation may prompt individuals to revise their personal fertility expectations if it represents a constraint to fertility. However, imaginaries about the future may remain unaltered as reflected by the lack of change in desires—a proxy for family imaginaries (Vignoli et al., 2020b). This distinction has important implications for understanding people’s fertility behaviours: individuals may continue to pursue their imagined future, despite having lower expectations for children, hence ‘deviating’ from what would be predicted based solely on expectations.

## Supplementary Material

The online version contains supplementary material available at https://doi.org/10.1186/s41118-025-00248-1.

Supplementary Material

## Figures and Tables

**Table 1:** Percentage distribution of demographic, socioeconomic, and background characteristics in T1

	Women				Men		
	Total	Fertile	Infertile		Total	Fertile	Infertile
Self-reported infertility							
Yes	14.2				6.1		
*Demographic characteristics*							
Age group							
18–24	27.8	29.6	17.1		24.3	25.3	9.2
25–29	20.2	20.5	18.4		18.5	18.8	14.9
30–34	16.3	16.4	15.4		15.6	15.6	15.9
35–39	12.9	12.5	15.2		12.2	12.1	14.4
40–44	12.3	11.5	16.9		12.8	12.3	21.5
45–49	10.6	9.6	16.9		9.4	9.0	15.9
50–54 (men only)					7.2	7.1	8.2
Parity							
0	51.3	52.1	46.1		56.4	57.7	36.9
1	15.7	14.9	20.7		14.8	14.1	25.6
2	20.9	20.8	21.1		17.4	17.1	22.1
3 and above	12.2	12.2	12.1		11.4	11.2	15.4
Relationship status							
Single	39.9	40.8	34.5		41.7	43.6	12.3
Cohabiting	24.5	24.3	25.4		23.8	23.5	28.7
Married	35.6	34.8	40.2		34.5	32.9	59.0
*Socio-economic characteristics*							
Education							
Low	34.8	34.6	36.4		39.1	39.7	29.2
Medium	29.8	29.5	31.5		34.4	34.0	40.0
High	35.4	36.0	32.1		26.6	26.3	30.8
Satisfaction with finances							
Very satisfied	16.2	17.0	11.6		16.5	16.7	12.8
Reasonably satisfied	52.2	53.2	45.9		51.3	51.8	43.6
Dissatisfied	31.6	29.8	42.5		32.2	31.5	43.6
*Background characteristics*							
Migration and indigenous status							
Non-migrant	55.2	54.8	57.9		54.1	53.7	60.5
Migrant	41.3	42.1	36.8		42.9	43.3	38.0
Indigenous Australian	3.5	3.2	5.3		3.0	3.0	1.5
Area of residence							
Major city	68.3	68.7	67.0		68.7	68.9	64.6
Inner regional	22.1	21.6	25.0		21.5	21.4	23.1
Outer regional, remote, or very remote	9.6	9.7	9.1		9.8	9.7	12.3

*N* = 3329 women and 3191 men interviewed in wave 15 with non-missing data on dependent and independent variables. Column percentage may not add to 100 because of rounding Source: HILDA survey, Wave 15, release 21

**Table 2:** OLS regression analyses of the association between self-reported infertility and fertility desires or expectations in T1

	Women						Men				
	Fertility desires			Fertility expectations			Fertility desires			Fertility expectations	
	Model 1	Model 2		Model 1	Model 2		Model 1	Model 2		Model 1	Model 2
Self-reported infertility											
No (Ref.)											
Yes	− 0.28	0.34*		− 1.44**	− 0.66**		− 0.85**	− 0.10		− 1.44**	− 0.60**
*Demographic characteristics*											
Age group											
18–24 (Ref.)											
25–29		− 0.31			− 0.53**			− 0.17			− 0.44*
30–34		− 1.27**			− 1.95**			− 0.96**			− 1.53**
35–39		− 2.89**			− 3.80**			− 2.23**			− 2.92**
40–44		− 4.25**			− 5.27**			− 3.96**			− 4.73**
45–49		− 4.97**			− 5.49**			− 4.18**			− 5.17**
50–54 (men only)								− 4.77**			− 5.50**
Parity											
0 (Ref.)											
1		− 0.23			− 0.34*			− 0.07			− 0.17
2		− 2.86**			− 2.89**			− 2.77**			− 2.75**
3 and above		− 3.48**			− 3.03**			− 2.89**			− 2.84**
Relationship status											
Single (Ref.)											
Cohabiting		0.64**			0.83**			0.83**			1.08**
Married		1.08**			1.12**			0.90**			1.03**
*Socio-economic characteristics*											
Education											
Lower (Ref.)											
Medium		0.02			0.11			0.19			0.33**
High		0.21			0.19			0.55**			0.59**
Satisfaction with finances											
Very satisfied		0.08			0.05			0.23			0.19
Reasonably satisfied (Ref.)											
Dissatisfied		− 0.05			− 0.29**			− 0.08			− 0.11
*Background characteristics*											
Migration and indigenous status											
Non-migrant (Ref.)											
Migrant		− 0.23*			− 0.11			0.29*			0.32**
Indigenous Australian		− 0.28			− 0.463			− 0.42			− 0.24
Area of residence											
Major city (Ref.)											
Inner regional		− 0.08			− 0.20			− 0.22			− 0.23
Outer regional or remote		− 0.20			− 0.17			− 0.08			− 0.15
Intercept	5.57**	7.75**		4.95**	7.51**		5.53**	7.31**		5.01**	7.03**

*N* = 3329 women and 3191 men interviewed in wave 15 with non-missing data on dependent and independent variables

* *p* < .05;

** *p* < .01

Source: HILDA survey, Wave 15, release 21

**Table 3:** Percentage distribution of demographic, socioeconomic, and background characteristics in T2

	Women	Men
Self-reported infertility at T2		
Yes	11.3	5.5
*Demographic characteristics*		
Age group at T1		
18–24	31.0	26.3
25–29	21.2	19.2
30–34	18.3	17.2
35–39	13.7	14.1
40–45	15.8	16.0
46–50 (men only)		7.1
Parity at T1		
0	53.9	58.4
1	15.8	15.0
2	19.4	16.8
3 and above	10.9	9.8
Had a birth between T1 and T2		
Yes	22.2	20.0
Change in relationship status between T1 and T2		
No change	83.6	84.7
From being in a union to single	4.9	4.2
From being single to being in a union	11.6	11.1
*Socio-economic characteristics*		
Education		
Low	34.8	38.4
Medium	28.5	34.6
High	36.8	27.0
Change in satisfaction with finances between T1 and T2		
No change (Ref.)	56.1	57.7
More satisfied	21.4	20.8
Less satisfied	22.5	21.6
*Background characteristics*		
Migration and indigenous status		
Non-migrant	55.4	53.4
Migrant	41.4	43.8
Indigenous Australian	3.2	2.8
Area of residence		
Major city	69.0	70.1
Inner regional	21.3	21.0
Outer regional, remote, or very remote	9.7	8.9

*N* = 1857 women and 1845 men interviewed in waves 15 and 19 with non-missing data on dependent and independent variables. Column percentage may not add to 100 because of rounding Source: HILDA survey, Waves 15 and 19, release 21

**Table 4:** OLS regression analyses predicting fertility desires (or expectations) in T2 from fertility desires (or expectations) in T1 and between-wave changes in self-reported infertility

	Women						Men				
	Fertility desires			Fertility expectations			Fertility desires			Fertility expectations	
	Model 1	Model 2		Model 1	Model 2		Model 1	Model 2		Model 1	Model 2
Self-reported infertility at T2											
No (Ref.)											
Yes	− 0.30	0.02		− 0.84**	− 0.54**		− 0.05	0.27		− 0.92**	− 0.58*
Fertility aspirations at T1^	0.69**	0.56**		0.67**	0.48**		0.66**	0.52**		0.66***	0.48**
*Demographic* *characteristics*											
Age group at T1											
18–24 (Ref.)											
25–29		− 0.50**			− 0.57**			− 0.33			− 0.42**
30–34		− 1.33**			− 1.63**			− 0.96**			− 1.34**
35–39		− 1.79**			− 2.10**			− 1.03**			− 1.60**
40–45		− 1.95**			− 2.00**			− 1.53**			− 1.99**
46–50 (men only)								− 1.72**			− 2.18**
Parity at T1											
0 (Ref.)											
1		− 1.74**			− 2.03**			− 1.75**			− 1.81**
2		− 1.59**			− 1.73**			− 1.81**			− 1.73**
3 and above		− 1.07**			− 1.43**			− 1.74**			− 1.60**
Had a birth between T1 and T2											
No (Ref.)											
Yes		− 1.21**			− 1.04**			− 1.20**			− 1.12**
Change in relationship status between T1 and T2											
No change (Ref.)											
From being in a union to single		− 0.05			− 0.16			− 0.31			− 0.35
From being single to being in a union		0.98**			1.25**			0.92**			1.12**
*Socio-economic characteristics*											
Education											
Low (Ref.)											
Medium		0.02			− 0.01			0.11			0.01
High		0.24			0.11			0.08			0.09
Change in satisfaction with finances between T1 and T2											
No change (Ref.)											
More satisfied		0.07			0.03			− 0.26			− 0.10
Less satisfied		− 0.02			− 0.27			− 0.31*			− 0.25
*Background characteristics*											
Migration and Indigenous status											
Non-migrant (Ref.)											
Migrant		0.02			0.07			0.26			0.20
Indigenous Australian		− 0.64			− 0.42			0.39			0.21
Area of residence											
Major city (Ref.)											
Inner regional		− 0.15			− 0.17			− 0.10			− 0.06
Outer regional or remote		0.05			0.06			− 0.05			− 0.05
Intercept	0.60**	3.02**		0.56**	3.48**		0.88**	3.25**		0.64**	3.34**

*N* = 1857 women and 1845 men interviewed in wave 15 and 19 with non-missing data on dependent and independent variables

* *p* < .05;

** *p* < .01.

^ Refers to fertility desires at T1 for the models predicting fertility desires at T2 and to fertility expectations at T1 for models predicting fertility expectations at T2

Source: HILDA survey, Wave 15 and Wave 19, release 21

**Table 5:** OLS regression analyses predicting fertility desires (or expectations) in T1 from fertility desires (or expectations) in T2 and between-wave changes in self-reported infertility, couple sample

	Women						Men				
	Fertility desires			Fertility expectations			Fertility desires			Fertility expectations	
	Model 1	Model 2		Model 1	Model 2		Model 1	Model 2		Model 1	Model 2
Self-reported infertility at T2											
No (Ref.)											
Yes	0.17	0.37		− 0.72*	− 0.55*		− 0.31	0.11		− 0.44	− 0.70*
Fertility aspirations at T1^	0.50**	0.47**		0.39**	0.33**		0.47**	0.41**		0.34**	0.27**
Partner’s fertility aspirations at T1^	0.16**	0.11**		0.21**	0.14**		0.16**	0.14**		0.28**	0.24**
*Demographic* *characteristics*											
Age group at T1											
18–24 (Ref.)											
25–29		− 0.40			− 0.50			− 0.35			− 0.46
30–34		− 1.22**			− 1.58**			− 1.20**			− 1.49**
35–39		− 1.42**			− 1.80**			− 1.04*			− 1.39**
40–45		− 1.67**			− 1.75**			− 1.42**			− 1.60**
46–50 (men only)								− 1.78**			− 1.83**
Age gap in the couple											
No gap or ± 3 years (Ref.)											
More than 3 years		− 0.20			− 0.34+			0.25			− 0.02
Parity at T1											
0 (Ref.)											
1		− 2.67**			− 2.89**			− 2.22**			− 2.46**
2		− 1.97**			− 2.21**			− 2.38**			− 2.41**
3 and above		− 1.39**			− 1.87**			− 2.01**			− 2.04**
Had a birth between T1 and T2											
No (Ref.)											
Yes		− 1.46**			− 1.20**			− 1.48**			− 1.50**
Relationship status											
Married (Ref.)											
Cohabiting		− 0.14			− 0.41*			0.33			0.53*
*Relationship satisfaction at T1*		− 0.06			− 0.09+			− 0.06			− 0.04
*Socio-economic characteristics*											
Education											
Both below tertiary (Ref.)											
Only woman tertiary		0.17			0.20			0.24			0.11
Only man tertiary		0.41			0.10			− 0.09			0.04
Both tertiary		0.69*			0.43			0.40			0.27
Change in satisfaction with finances between T1 and T2											
No change (Ref.)											
More satisfied		0.21			0.12			− 0.17			− 0.07
Less satisfied		− 0.13			− 0.50*			− 0.17			− 0.26
Background characteristics											
Migration and indigenous status											
Non-migrant (Ref.)											
Migrant		0.13			− 0.01			0.65**			0.43*
Indigenous Australian		− 0.36			− 0.34*			0.88			0.97
Partner’s migration and indigenous status											
Non-migrant (Ref.)											
Migrant		0.29			0.42			− 0.33			− 0.20
Indigenous Australian		1.08+			1.11*			− 0.10			0.11
Area of residence											
Major city (Ref.)											
Inner regional		− 0.07			− 0.16			− 0.15			− 0.20
Outer regional or remote		− 0.24			0.02			− 0.42			− 0.23
Intercept	0.08	3.35**		0.09	4.21**		0.31	3.90**		0.10	3.90**

*N* = 777 couples interviewed in wave 19 with non-missing data on dependent and independent variables

* *p* < .05;

** *p* < .01.

^ Refers to fertility desires at T1 for the models predicting fertility desires at T2 and to fertility expectations at T1 for models predicting fertility expectations at T2 Source: HILDA survey, Wave 19, release 21

## Data Availability

The data that support the findings of this study are available to approved researchers from government, academic institutions, and non-profit organizations and accessible from the Australian Data Archive upon application (https://dataverse.ada.edu.au/dataverse/ada).

## Declarations

1 The TDI model makes an explicit distinction between expectations and intentions, in that intentions only consider situational factors while expectations factor in all external constraints beyond the individual’s direct influence. By contrast, the CSM of fertility intentions suggests that situational constraints are incorporated into the formation of intentions, making the distinction between the two unnecessary (Bachrach and Morgan 2013).

2 Individuals who identified as infertile in T1 had a slightly lower retention rate at follow-up (74.4%) compared to those who did not identify as infertile (77.3%). However, results from a Chi-square test indicated that this difference was not statistically significant (*p* > 0.05), suggesting that selective attrition is unlikely to bias the findings.

## Abbreviations

ART: Assisted reproductive technology
HILDA: Household Income and Labour Dynamics in Australia Survey
T1: Time 1
T2: Time 2
TCA: Theory of conjunctural action
TDI: Traits–desires–intentions
TPB: Theory of planned behaviour
