# Supplementary material for: Fertility preferences adjusted: reimagining parenthood in response to the uncertainty of infertility
Source: Genus. Author manuscript; Available in PMC 2025 May 19. (PMC7617686; doi:10.1186/s41118-025-00248-1)
Supplement: Supplementary Material [file EMS205465-supplement-Supplementary_Material.docx]

**Table S1**. Means and distributions of fertility desires and expectations in T2 by self-reported infertility status.

|  | Women | | Men | |
| --- | --- | --- | --- | --- |
|  | Fertile | Infertile | Fertile | Infertile |
| Fertility desires (mean) | 5.2 | 4.8 | 5.3 | 4.8 |
| Score distribution (percent) |  |  |  |  |
| 0 | 26.6 | 33.6 | 22.9 | 33.2 |
| 1 | 4.5 | 3.2 | 4.3 | 4.6 |
| 2 | 4.1 | 5.4 | 3.6 | 1.8 |
| 3 | 4.1 | 2.6 | 4.0 | 4.3 |
| 4 | 1.6 | 2.0 | 2.2 | 1.5 |
| 5 | 10.0 | 7.0 | 9.2 | 6.8 |
| 6 | 3.2 | 2.6 | 5.7 | 4.9 |
| 7 | 7.2 | 6.0 | 9.2 | 5.3 |
| 8 | 7.8 | 7.8 | 12.2 | 9.3 |
| 9 | 6.8 | 6.0 | 7.9 | 5.5 |
| 10 | 24.1 | 23.8 | 18.9 | 22.8 |
|  |  |  |  |  |
| Fertility expectations (mean) | 4.6 | 3.3 | 4.9 | 3.4 |
| Score distribution (percent) |  |  |  |  |
| 0 | 30.8 | 40.0 | 24.2 | 39.8 |
| 1 | 6.2 | 7.8 | 7.0 | 8.8 |
| 2 | 3.6 | 6.2 | 4.9 | 4.2 |
| 3 | 3.3 | 3.8 | 3.5 | 2.3 |
| 4 | 1.8 | 3.3 | 1.8 | 4.0 |
| 5 | 11.3 | 11.8 | 11.2 | 10.5 |
| 6 | 3.3 | 5.0 | 5.4 | 3.2 |
| 7 | 6.2 | 4.9 | 8.5 | 9.3 |
| 8 | 9.7 | 4.9 | 9.8 | 4.2 |
| 9 | 6.9 | 3.9 | 9.8 | 4.3 |
| 10 | 17.0 | 8.5 | 14.0 | 9.6 |

Note: N= 3,464 women and 3,334 men interviewed in wave 19 with non-missing data on dependent and independent variables. Column percentage may not add to 100 because of rounding. Weighted.

Source: HILDA survey, wave 19, release 21.

**Table S2**. Percentage distribution of demographic, socio-economic and background characteristics at T2.

|  | Women | | | Men | | |
| --- | --- | --- | --- | --- | --- | --- |
| *Self-reported infertility* | Total | Fertile | Infertile | Total | Fertile | Infertile |
| Yes | 16.1 |  |  | 7.0 |  |  |
| **Demographic characteristics** |  |  |  |  |  |  |
| *Age group* |  |  |  |  |  |  |
| 18-24 | 24.5 | 25.9 | 17.2 | 23.6 | 24.7 | 8.6 |
| 25-29 | 20.7 | 21.2 | 18.1 | 19.2 | 19.2 | 19.0 |
| 30-34 | 19.3 | 19.3 | 19.4 | 17.6 | 17.3 | 22.4 |
| 35-39 | 13.7 | 13.8 | 13.1 | 13.5 | 13.4 | 15.1 |
| 40-44 | 10.6 | 10.1 | 13.3 | 10.7 | 10.5 | 12.5 |
| 45-49 | 11.3 | 9.8 | 19.0 | 10.1 | 9.6 | 16.4 |
| 50-54 (Men only) |  |  |  | 5.5 | 5.4 | 6.0 |
| *Parity* |  |  |  |  |  |  |
| 0 | 50.1 | 51.0 | 45.0 | 55.8 | 56.6 | 45.3 |
| 1 | 15.6 | 14.3 | 22.8 | 13.7 | 13.0 | 23.7 |
| 2 | 22.2 | 22.5 | 20.8 | 19.5 | 19.3 | 22.8 |
| 3 and above | 12.1 | 12.2 | 11.5 | 10.9 | 11.1 | 8.2 |
| *Relationship status* |  |  |  |  |  |  |
| Single | 38.3 | 38.2 | 38.7 | 41.2 | 43.3 | 12.5 |
| Cohabiting | 27.2 | 27.7 | 36.9 | 25.6 | 25.1 | 32.8 |
| Married | 34.6 | 34.1 | 24.4 | 33.2 | 31.6 | 54.7 |
| **Socio-economic characteristics** |  |  |  |  |  |  |
| *Education* |  |  |  |  |  |  |
| Low | 32.2 | 32.3 | 30.5 | 38.2 | 38.6 | 33.6 |
| Medium | 28.6 | 28.5 | 35.8 | 35.1 | 34.6 | 41.8 |
| High | 39.2 | 39.2 | 33.7 | 26.7 | 26.9 | 24.6 |
| *Satisfaction with finances* |  |  |  |  |  |  |
| Very satisfied | 16.8 | 17.6 | 12.7 | 18.2 | 18.5 | 16.4 |
| Reasonably satisfied | 51.2 | 52.2 | 46.2 | 50.5 | 50.6 | 50.4 |
| Dissatisfied | 32.0 | 30.2 | 41.0 | 31.3 | 30.9 | 33.2 |
| **Background characteristics** |  |  |  |  |  |  |
| *Migration and Indigenous status* |  |  |  |  |  |  |
| Non-migrant | 55.9 | 55.7 | 57.0 | 54.8 | 54.4 | 59.5 |
| Migrant | 40.1 | 40.3 | 39.3 | 41.5 | 41.7 | 38.8 |
| Indigenous Australian | 4.0 | 4.0 | 3.8 | 3.8 | 3.9 | 1.7 |
| *Area of residence* |  |  |  |  |  |  |
| Major city | 66.5 | 67.3 | 62.4 | 66.6 | 67.0 | 60.8 |
| Inner regional | 23.5 | 22.8 | 27.2 | 23.9 | 23.5 | 29.7 |
| Outer regional, remote, or very remote | 9.9 | 9.8 | 10.4 | 9.5 | 9.5 | 10.4 |

Note: N= 3,464 women and 3,334 men interviewed in wave 19 with non-missing data on dependent and independent variables. Column percentage may not add to 100 because of rounding.

Source: HILDA survey, wave 19, release 21.

**Table S3**. OLS regression analyses of the association between self-reported infertility and fertility desires or expectations at T2.

|  | Women | | | | Men | | | |
| --- | --- | --- | --- | --- | --- | --- | --- | --- |
|  | Fertility desires | | Fertility expectations | | Fertility desires | | Fertility expectations | |
|  | Model 1 | Model 2 | Model 1 | Model 2 | Model 1 | Model 2 | Model 1 | Model 2 |
| *Self-reported infertility* |  |  |  |  |  |  |  |  |
| No (Ref.) |  |  |  |  |  |  |  |  |
| Yes | -0.47* | 0.13 | -1.41*** | -0.73*** | 0.01 | 0.22 | -0.94*** | -0.64*** |
| **Demographic characteristics** |  |  |  |  |  |  |  |  |
| *Age group* |  |  |  |  |  |  |  |  |
| 18-24 (Ref.) |  |  |  |  |  |  |  |  |
| 25-29 |  | -0.50** |  | -0.69*** |  | -0.01 |  | -0.22 |
| 30-34 |  | -1.59*** |  | -1.96*** |  | -0.81*** |  | -1.14*** |
| 35-39 |  | -2.95*** |  | -3.80*** |  | -2.04*** |  | -2.64*** |
| 40-44 |  | -4.42*** |  | -5.13*** |  | -2.94*** |  | -3.83*** |
| 45-49 |  | -5.37*** |  | -5.73*** |  | -4.10*** |  | -4.73*** |
| 50-54 (Men only) |  |  |  |  |  | -4.31*** |  | -5.05*** |
| *Parity* |  |  |  |  |  |  |  |  |
| 0 (Ref.) |  |  |  |  |  |  |  |  |
| 1 |  | -0.07 |  | -0.29† |  | -0.08 |  | -0.04 |
| 2 |  | -2.50*** |  | -2.41*** |  | -2.76*** |  | -2.61*** |
| 3 and above |  | -2.68*** |  | -2.58*** |  | -3.23*** |  | -2.91*** |
| *Relationship status* |  |  |  |  |  |  |  |  |
| Single (Ref.) |  |  |  |  |  |  |  |  |
| Cohabiting |  | 0.78*** |  | 1.00*** |  | 0.69*** |  | 0.95*** |
| Married |  | 1.08*** |  | 1.02*** |  | 0.95*** |  | 0.91*** |
| **Socio-economic characteristics** |  |  |  |  |  |  |  |  |
| *Education* |  |  |  |  |  |  |  |  |
| Low (Ref.) |  |  |  |  |  |  |  |  |
| Medium |  | 0.13 |  | 0.12 |  | 0.14 |  | 0.07 |
| High |  | 0.56*** |  | 0.49*** |  | 0.19 |  | 0.17 |
| *Satisfaction with finances* |  |  |  |  |  |  |  |  |
| Very satisfied |  | 0.11 |  | 0.07 |  | 0.27† |  | 0.35** |
| Reasonably satisfied (Ref.) |  |  |  |  |  |  |  |  |
| Dissatisfied |  | -0.18 |  | -0.27* |  | -0.07 |  | -0.22† |
| **Background characteristics** |  |  |  |  |  |  |  |  |
| *Migration and Indigenous status* |  |  |  |  |  |  |  |  |
| Non-migrant (Ref.) |  |  |  |  |  |  |  |  |
| Migrant |  | -0.02 |  | 0.11 |  | 0.28* |  | 0.22* |
| Indigenous Australian |  | -0.19 |  | -0.24 |  | -0.06 |  | -0.24 |
| *Area of residence* |  |  |  |  |  |  |  |  |
| Major city (Ref.) |  |  |  |  |  |  |  |  |
| Inner regional |  | -0.31* |  | -0.33** |  | -0.44*** |  | -0.43*** |
| Outer regional or remote |  | -0.11 |  | -0.15 |  | -0.04 |  | -0.02 |
|  |  |  |  |  |  |  |  |  |
| *Intercept* | 5.21*** | 7.20*** | 4.71*** | 7.00*** | 5.26*** | 6.90*** | 4.82*** | 6.80*** |

Note: N= 3,464 women and 3,334 men interviewed in wave 19 with non-missing data on dependent and independent variables.

†p < .10; *p < .05; **p < .01; ***p < .001

Source: HILDA survey, wave 19, release 21.

**Table S4**. Means and distributions of fertility desires and expectations in T1 by self-reported infertility status.

|  | Women | | Men | |
| --- | --- | --- | --- | --- |
|  | Fertile | Infertile | Fertile | Infertile |
| Fertility desires (mean) | 5.5 | 5.0 | 5.5 | 4.2 |
| Score distribution (percent) |  |  |  |  |
| 0 | 24.3 | 31.6 | 27.2 | 41.7 |
| 1 | 5.6 | 4.8 | 7.8 | 9.5 |
| 2 | 3.7 | 2.7 | 4.3 | 6.1 |
| 3 | 3.2 | 2.4 | 3.4 | 2.3 |
| 4 | 2.3 | 0.9 | 2.5 | 1.4 |
| 5 | 6.8 | 8.0 | 7.8 | 12.8 |
| 6 | 3.8 | 3.7 | 3.2 | 4.3 |
| 7 | 7.2 | 6.1 | 6.9 | 6.3 |
| 8 | 9.1 | 7.0 | 8.7 | 5.9 |
| 9 | 6.2 | 7.2 | 8.2 | 2.4 |
| 10 | 27.9 | 25.6 | 20 | 7.4 |
|  |  |  |  |  |
| Fertility expectations (mean) | 4.9 | 3.1 | 5.0 | 3.0 |
| Score distribution (percent) |  |  |  |  |
| 0 | 23.1 | 37.9 | 24.5 | 44.8 |
| 1 | 4.9 | 4.8 | 7.1 | 6.7 |
| 2 | 2.8 | 6.1 | 4.3 | 9.3 |
| 3 | 3.1 | 0.7 | 3.7 | 2.7 |
| 4 | 1.9 | 1.3 | 2.3 | 2.4 |
| 5 | 8.7 | 9.5 | 9.6 | 8.4 |
| 6 | 4.9 | 3.0 | 4.4 | 2.2 |
| 7 | 8.6 | 6.2 | 7.9 | 5.7 |
| 8 | 11.1 | 7.3 | 10.2 | 7.9 |
| 9 | 7.3 | 4.4 | 8.1 | 2.2 |
| 10 | 23.7 | 18.9 | 18.1 | 8.0 |

Note: N= 3,329 women and 3,191 men interviewed in wave 19 with non-missing data on dependent and independent variables. Column percentage may not add to 100 because of rounding. Weighted.

Source: HILDA survey, wave 15, release 21.

**Table S5**. Mean change in fertility desires and expectations between T1 and T2 for respondents who were fertile in T1 by their infertility status in T2.

|  | Infertility status at T2 | | | |
| --- | --- | --- | --- | --- |
|  | Women | | Men | |
|  | Fertile | Infertile | Fertile | Infertile |
| Fertility desires at T1 | 5.81 | 5.72 | 5.82 | 6.38 |
| Fertility desires at T2 | 4.62 | 4.26 | 4.69 | 5.01 |
| Fertility expectations at T1 | 5.16 | 4.90 | 5.30 | 5.75 |
| Fertility expectations at T2 | **4.03** | **3.01** | 4.14 | 3.51 |

Note: N= 1,857 women and 1,845 men interviewed in wave 15 and 19 with non-missing data on dependent and independent variables. Bold numbers highlight statistically significant differences (p < 0.05) between respondents who self-identified as fertile and infertile at T2, as determined by a two-sample t-test.

Source: HILDA survey, wave 15 and 19, release 21.
